# Neurocognition and social cognition in patients with schizophrenia spectrum disorders with and without a history of violence: results of a multinational European study

**DOI:** 10.1038/s41398-021-01749-1

**Published:** 2021-12-08

**Authors:** Laura Iozzino, Philip D. Harvey, Nicola Canessa, Pawel Gosek, Janusz Heitzman, Ambra Macis, Marco Picchioni, Hans Joachim Salize, Johannes Wancata, Marlene Koch, Clarissa Ferrari, Giovanni de Girolamo

**Affiliations:** 1 Unit of Epidemiological Psychiatry and Evaluation, IRCCS Istituto Centro San Giovanni di Dio Fatebenefratelli, Brescia, Italy; 2 Department of Psychiatry and Behavioral Sciences; Research Service, University of Miami Miller School of Medicine, Miami, FL USA; 3 Scuola Universitaria Superiore IUSS, IUSS Cognitive Neuroscience (ICoN) Center, Pavia, Italy; 4 Cognitive Neuroscience Laboratory of Pavia Institute, Istituti Clinici Scientifici Maugeri IRCCS, Pavia, Italy; 5 Department of Forensic Psychiatry, Institute of Psychiatry and Neurology, Warsaw, Poland; 6 Unit of Statistics, IRCCS Istituto Centro San Giovanni di Dio Fatebenefratelli, Brescia, Italy; 7 Department of Forensic and Neurodevelopmental Science, Institute of Psychiatry, Psychology and Neuroscience, King’s College London, London, UK; 8 St Magnus Hospital, Haslemere, Surrey UK; 9 Medical Faculty Mannheim/Heidelberg University, Central Institute of Mental Health Mannheim, Mannheim, Germany; 10 Clinical Division of Social Psychiatry, Medical University of Vienna, Vienna, Austria

**Keywords:** Schizophrenia, Human behaviour

## Abstract

**Objective:**

Neurocognitive impairment has been extensively studied in people with schizophrenia spectrum disorders and seems to be one of the major determinants of functional outcome in this clinical population. Data exploring the link between neuropsychological deficits and the risk of violence in schizophrenia has been more inconsistent. In this study, we analyse the differential predictive potential of neurocognition and social cognition to discriminate patients with schizophrenia spectrum disorders with and without a history of severe violence.

**Methods:**

Overall, 398 (221 cases and 177 controls) patients were recruited in forensic and general psychiatric settings across five European countries and assessed using a standardized battery.

**Results:**

Education and processing speed were the strongest discriminators between forensic and non-forensic patients, followed by emotion recognition. In particular, increased accuracy for anger recognition was the most distinctive feature of the forensic group.

**Conclusions:**

These results may have important clinical implications, suggesting potential enhancements of the assessment and treatment of patients with schizophrenia spectrum disorders with a history of violence, who may benefit from consideration of socio-cognitive skills commonly neglected in ordinary clinical practice.

## Introduction

It is a general impression that people with schizophrenia spectrum disorders (SSD) can be violent or dangerous. Generally, however, violence on the part of people with SSD is no more common than in the general population in similar neighbourhoods [1, 2]. Violence does have several different features compared to violence in the general population: it is less commonly financially motivated and can be unpredictable and directed toward strangers [3]. Most victims of aggressive behaviour on the part of people with SSD are actually family members, fellow patients and mental health professionals injured while attempting to treat people with SSD. This may also not be different from the general population. In a recent study, for example, among male homicide victims, only 29% were killed by someone they did not know and assaults were committed by someone known to the victim in 64% of cases [4].

Aggressive and violent behaviour, including both verbal and physical aggression, have considerable adverse consequences for people with SSD. In several countries most psychiatric hospital beds are occupied by forensic patients with SSD. The primary reasons preventing discharge are, in order of importance: impulsivity, hostility, excitement and uncooperativeness. As a result, individuals with specific treatment-resistant symptoms despite pharmacological therapies prevent safe discharges even when the hospital management is highly motivated to move patients to community settings [5]. Verbal aggression is also a barrier to successful discharge from long-term care, so actual physical violence is not required to prevent discharge to less restrictive settings [6].

Based on correlational studies, there are several potential causes of violent behaviour in people with SSD: these include cognitive and social cognitive deficits, functional skills deficits, substance use disorders and the emotional response to specific psychotic features. Although cognitive impairments are common in SSD and are clearly correlated with many elements of disability, the research on cognitive impairments as a determinant of violence has also yielded some important findings: prototypical cognitive impairments seen in SSD are less strongly implicated for causing violence than for leading to deficits in everyday functional skills.

With regard to social cognition (SC), this includes several different domains, including understanding others’ mental states, recognizing emotions and making attributions for the reasons that others act the way that they do [7]. These elements of SC diverge in their functional importance. Understanding mental states and recognizing emotions appear to be related to impairments in everyday social functioning, while attributions fail to predict these types of disability. In particular, previous studies have shown deficits in facial affect recognition in patients with aggressive behaviour and impaired recognition of fear and anger, alongside misjudgement of neutral expressions as indicative of fear or anger, are characteristic of patients displaying violent behaviour [8]. However, attribution style predicts the presence of paranoid ideation [9], which, particularly when severe delusions are present, is associated with unprovoked attacks on others [10]. Thus, attributional style may be a contributor to believing that others are mistreating you, leading to attempts to contravene and reduce threats.

Given the fact that both cognitive and social cognitive deficits have been reported to be associated with violence in SSD, it would be informative to understand their differential contributions. Some previous studies have shown that social cognitive performance is less discriminating between healthy controls (HC) and patients with SSD than neurocognitive performance [11], while others have found that social cognitive deficits were relatively greater [12], and that social cognitive impairments seem to be more proximally related to aggressive behaviour than neurocognitive deficits [13, 14]. Further, SC and neurocognition manifest some correlational overlap. In a previous study [15] the authors examined the multivariate ability of SC tests to discriminate HC and SSD and the importance of neurocognitive performance as a covariate. The overall effect of diagnosis on social cognitive measures was significant (*p* < 0.001). The covariate effect of the composite cognitive was also significant (*p* < 0.001). There is evidence, however, that violent behaviour is better predicted by domain-specific cognitive alterations (involving for instance working memory, reasoning/problem solving and verbal learning) than by super-ordinate IQ measures [16], which highlights the need of an in-depth characterization of neuro-cognitive functioning in forensic populations.

### Aims of the study

In the present study, we examine the differential predictive potential of neurocognition and SC to discriminate violent forensic vs non-violent participants with SSD in a large-scale multinational study. As both of these domains have independently been associated with violence and aggression [14, 16], it is possible that both contribute to these distinctions. Further, treatment of both neurocognition and SC have been found to reduce violence, in both general population and forensic patients [17, 18]. Finally, combining SC and neurocognition training leads to greater treatment gains, even in very chronic patients [19, 20].

Our hypotheses were that both SC and neurocognition would be more impaired in the forensic patients. We further hypothesized that attributions and emotional recognition would be the most important social cognitive domains to differentiate forensic status. Finally, we hypothesized that both deficits in neurocognition and in SC would provide good discrimination of these two participant populations.

## Methods

### Participants

EU-VIORMED is a European multicentre observational study [21]. The field work was conducted in five European countries: Austria, Germany, Italy, Poland and the United Kingdom. All subjects were between 18 and 65 years of age with a primary DSM-5 diagnosis of an SSD [22]. “Cases” were patients with a primary diagnosis of an SSD and a history of significant interpersonal violence. They were recruited from multiple forensic services in each country (see Table 1S in Supplementary files). Significant interpersonal violence was defined as having committed a homicide, attempted homicide or other assault that caused serious physical injury to another person. “Controls” were sex and age-matched patients with SSDs who have never committed such an act of violence and were recruited from general psychiatric services. Exclusion criteria included: (i) a confirmed intellectual disability; (ii) a traumatic brain injury or organic brain disorders; (iii) not being able to speak the national language fluently; and (iv) planned discharge from psychiatric services in the next month.

Initial plans were to recruit 200 cases and 200 gender- and age-matched controls. However, the worldwide coronavirus outbreak and the resulting restrictions from February 2020 caused recruitment to temporarily halt in every country. Once recruitment restarted, some restrictions remained and it was more feasible to over-recruit forensic cases rather than controls.

The study was approved by the Research Ethics Committee for the coordinating Centre (IRCCS Centro San Giovanni di Dio Fatebenefratelli, Brescia, Italy: no. 74-2018), and by the relevant Research Ethics Committees for each of the participating sites (listed at the end of the paper). All participants provided written informed consent before entering the study.

### Measures

All subjects were evaluated by research assistants employed by the study and centrally trained on each instrument. Socio-demographic, core clinical and criminological and violence risk data were collected using a study-specific Patient Information Form (PIF), an Index Violence Sheet (IVS) and a Risk Factors Questionnaire (RFQ) based on patient interviews later cross-referenced with the medical records and clinical reviews. DSM-5 diagnoses were based on clinicians’ evaluations extracted from the medical records.

Current psychotic symptoms were assessed using the Positive and Negative Syndrome Scale (PANSS) [23], based on a semi-structured patient interview and clinical observation. PANSS scoring used the original standard PANSS model; the PANSS overall total score ranges from 30 to 210. All research assistants underwent official centralized PANSS training provided by the PANSS Institute and were certified PANSS raters.

The World Health Organization Disability Assessment Schedule 2.0 (WHODAS 2.0) [24] was used to assess day-to-day functioning across six functional domains: cognition, mobility, self-care, getting along, life activities and participation. Scores were calculated using a simple sum, yielding a total from 0 to 48, with higher scores indicating more severe problems.

### Cognitive assessment

The Brief Assessment of Cognition in Schizophrenia (BACS) [25, 26] is a paper-and-pencil standardized neuropsychological instrument used to evaluate cognitive impairments and their relationship with functional outcomes in patients with schizophrenia. It includes six tests measuring different cognitive constructs: verbal (list learning) and working (Digit Sequencing Task) memory, motor speed (Token Motor Task), verbal fluency (semantic and letter fluency), attention and speed information processing (Symbol Coding Task) and executive functions (Tower of London).

### Social cognition: emotion recognition task (ER)

ER accuracy was measured using an Emotion Recognition task based on the Radboud Faces Database-RaFD (http://www.socsci.ru.nl:8180/RaFD2/RaFD) [27]. The RaFD includes high-quality pictures of models (including Caucasian males and females) depicting eight emotional expressions (anger, disgust, fear, happiness, sadness, surprise, contempt and neutral) that can be used freely for non-commercial scientific research. The Emotion Recognition task was administered by paper and pencil due to the prohibition to introduce computer or tablet in the forensic units. The adapted version of the test includes a set of 80 male and female pictures (40 + 40, i.e., ten per each picture type). Each picture was displayed for 5 s, and subjects were asked to name the emotion shown. Based on the number of correct responses, the overall ER score can range from 0 to 80, while the accuracy for each emotion ranges from 0 to 10. We used the overall ER score as measure of ER performance. We also calculated the misidentification scores for each of the eight emotions. Following previous studies [28], we defined misidentification as the tendency to confuse different emotions, for example, labelling happiness as fear. In particular, we focused on the misidentification of anger from other emotions to check for the presence of a hostile misattribution bias on ER performance.

### Social cognition: story-based empathy task (SET)

The SET is a non-verbal task developed by Dodich et al. [29] and is based on original cartoons. It lasts 15/20 min and consists of two main experimental conditions, i.e., identifying intentions (SET-IA) and emotional states (SET-EA), plus a control condition entailing the inference of causality reaction based on the knowledge of the physical properties of objects and human bodies (SET-CI). Each condition includes six trials requiring to select the correct ending of a comic strip. An upper (story) and a lower row of three vignettes (possible endings) compose each comic strip. To ensure subjects’ intact comprehension of the instructions, subjects were initially required to describe the story and to formulate a possible story ending, by presenting them only the upper vignettes without the possible endings. The possible endings are then presented after exposure to the initial story. A score of 1 is assigned when the correct ending is provided/selected, and the global score is computed based on the number of correct answers given by the subjects for each cartoon, leading to a possible global score (GS) up to 18. Each condition has a maximum score of 6 points. A “trial” run preceded the task, consisting of an example of causal attribution that would not appear in the testing phase.

### Statistical analyses

Frequencies and percentages for categorical variables and means and standard deviations for continuous variables were evaluated. Chi-square or Fisher’s exact tests were used to compare the categorical variables between the groups. The normality assumption for the distribution of the continuous variables was established by histogram plots and normality tests; depending on those results group comparisons were performed by *t*-tests (with Levene’s test for the equality of variances) or the non-parametric Mann–Whitney tests. Cohen’s *d* effect size was computed for evaluating the magnitude of the group mean differences (*d* ≤ 0.2 small effect size; 0.2 < *d* ≤ 0.5 small-medium; 0.5 < *d* ≤ 0.8 medium-large; *d* ≥ 0.8 very large effect size). Linear and generalized linear models were applied to assess the association between groups and neurocognitive and social cognition variables allowing the adjustment for potential confounders.

BACS *z*-scores have been obtained by using USA normative data [30]. Correlations among education, BACS, ER and SET scores have been evaluated using the Spearman coefficient rho (*ρ*). Moreover, univariate and multiple logistic models were performed to investigate the association between SC variables (independent variables) and the two groups (forensic and control group), unadjusted and then adjusted for BACS composite score, gender and education. Finally, a partial least-squares discriminant analysis (PLS-DA) has been performed to identify the variables (covariates) that best discriminate between the two groups. PLS-DA is a technique that allows to perform variable selection and classification in a one-step procedure; in particular, this technique allows to obtain for each covariate, independently of others, a loading representing the weight of each variable in discriminating between the two groups. Further investigation for the ER test regarding the misidentification pattern of its tasks was analysed by binomial proportion test.

The percentage of missing data was below 10% for all the variables included in the analyses, except for ER and SET scales, for which the percentage reached values around 15%. All missing data were evaluated to be missing at random (MAR).

All tests were two-side, and the significance level was set at 0.05. The analyses have been performed using IBM SPSS Statistics for Windows (Version 26.0., IBM Corp., Armonk, NY) and software R (R Core Team, 2020, version 4.0.3). In particular, R software was used for the logistic models, for correlation analysis (packages *Hmisc* and *corrplot*) and for the PLS-DA (package *mixOmics)*.

The power analysis and the computation of the sample size had been exhaustively described in the study protocol [21].

## Results

### Sociodemographic and clinical characteristics

Of 575 patients invited to join the study, 175 refused (99 cases, 30.9% and 76 controls, 30.0%). Cases and controls’ refusals differed significantly between the five countries (*p* = 0.002 and *p* < 0.001, respectively). In particular, cases’ refusal rates in Poland were lower than the other countries, while controls’ refusal rate was higher in Germany and in Poland than the other countries.

The final sample included 398 patients with a primary diagnosis of SSDs: 221 cases had a lifetime history of serious interpersonal violence and 177 controls without this history. The two groups did not differ in age (*p* = 0.291), and most subjects were males (*N* = 336; 84.4%), with a further male excess in the forensic sample (*p* = 0.019): thus, all subsequent analyses were adjusted for gender. Cases and controls did not differ on marital and occupational status (Table 1), but cases had lower educational achievement than controls (*p* < 0.001).

**Table 1:** Socio-demographic and clinical characteristics of forensic patients with SSD and controls.

	Forensic group *N* = 221 *N* (%)	Control group *N* = 177 *N* (%)	*P* value
**Sex**			
Male	195 (88.2)	141 (79.7)	**0.019**
Female	26 (11.8)	36 (20.3)	
**Age**			
18–29	50 (22.6)	52 (29.4)	0.291
30–41	93 (42.1)	60 (33.9)	
42–53	45 (20.4)	40 (22.6)	
54–65	33 (14.9)	25 (14.1)	
**Marital status**			
Married or cohabiting	10 (4.5)	15 (8.5)	0.223
Single	183 (82.8)	144 (81.4)	
Divorced or widowed	28 (12.7)	18 (10.2)	
Education years, mean (SD)^a^	11.5 (3.3)	12.9 (3.4)	**<0.001**
	*d* = −0.42		
**Highest occupational status**^a^			
Never worked/student/housewife	32 (14.5)	25 (14.3)	0.427
Unskilled worker	114 (51.6)	77 (44.0)	
Skilled worker	64 (29.0)	63 (36.0)	
Professional	11 (5.0)	10 (5.7)	
Illness duration (years), mean (SD)^a^	13.2 (9.6)	13.7 (10.5)	0.635
	*d* = −0.05		
Age of first contact with DMHs (years), mean (SD)^a^	25.0 (9.l)	22.8 (8.1)	**0.013**
	*d* = 0.25		
**Type of SSD diagnosis**			
Schizophrenia	174 (78.7)	130 (73.4)	**<0.001**
Schizoaffective disorders	22 (10.0)	41 (23.2)	
Delusional disorder	12 (5.4)	1 (0.6)	
Brief psychotic disorder	1 (0.5)	1 (0.6)	
Schizophreniform disorder	5 (2.3)	1 (0.6)	
Drug-induced psychosis	7 (3.2)	3 (1.7)	
**Comorbidity with personality disorders**^**a**^			
No	152 (70.7)	159 (92.4)	**<0.001**
Yes	63 (29.3)	13 (7.6)	
**Lifetime substance use**^**a**^			
Never	50 (22.7)	46 (26.1)	0.432
Yes	170 (77.3)	130 (73.9)	
**Antipsychotics**^**a**^			
No	3 (1.4)	6 (3.5)	0.191
Yes	214 (98.6)	165 (96.5)	
**PANSS**, mean (SD)			
Positive symptoms^a^	14.8 (6.9)	15.6 (5.7)	**0.020**
	*d* = −0.13		
Negative symptoms^a^	18.9 (7.7)	18.3 (6.5)	0.789
	*d* = 0.08		
General psychopathology^a^	33.9 (11.2)	34.5 (9.2)	0.121
	*d* = −0.06		
Total score^a^	67.8 (23.0)	68.5 (18.5)	0.226
	*d* = −0.03		
WHODAS 2.0, Total score mean (SD)^a^	8.0 (8.4)	12.8 (8.0)	**<0.001**
	*d* = −0.58		

*PANSS* Positive and Negative Syndrome Scale, *WHODAS 2.0* World Health Organization Disability Assessment Schedule 2.0.

^a^Frequencies and percentages (for categorical variables) and mean and standard deviations (for continuous variables) have been evaluated considering only the valid cases (i.e., all the cases with no missing data).

Chi squared or Fisher’s exact test (when expected count <5 in at least one cell) has been performed for categorical variables; *t*-test has been performed for Education years, Illness duration and age of first contact with DMHs; Mann–Whitney non-parametric test has been performed for all PANSS scores and for WHODAS 2.0 overall score.

*d*: Cohen’s *d* effect size (forensic group − control group; *d* ≤ 0.2 small effect size; 0.2 < *d* ≤ 0.5 small-medium; 0.5 < *d* ≤ 0.8 medium-large; *d* ≥ 0.8 very large effect size).

Bold values indicates statistically significant *p* values.

The most common primary diagnoses in both groups were schizophrenia (76.4%) and schizoaffective disorder (15.8%). Mean age at first contact with psychiatric services was significantly later in the case group (*p* = 0.013): however, the mean duration of illness was over 13 years in both groups. Cases were more likely to meet clinical criteria for a comorbid personality disorder than controls (*p* < 0.001). There were no differences between the two groups in lifetime substance use disorders (*p* = 0.432).

No significant differences were observed on the current PANSS total score between the two groups (*p* = 0.226) (Table 1), but controls had somewhat more severe current positive symptoms (mean score: 15.6, SD = 5.7 for controls vs mean score: 14.8, SD = 6.9 for cases; *p* = 0.020).

### Cognition and social cognition

To provide a reference for assessing SC skills in the clinical samples (forensic cases and non-forensic patients), emotion recognition accuracy was compared with the performance of a convenience sample of 57 healthy adults (32 females) recruited from acquaintances of researchers.

Both patient groups displayed worse emotion recognition skills compared with healthy individuals; however, only non-forensic patients had significant lower scores than healthy subjects after adjusting for gender and education. A closer inspection of these data show that non-forensic patients’ reduced performance was driven by their altered recognition of negative emotions such as fear and contempt (all *p* < 0.05). Moreover, we observed significant differences (and with moderate–high Cohen’s *d* effect size: all *d* larger than 0.33) across patient groups concerning their emotion recognition skills (Supplementary Tables 2S and 3S).

Forensic and non-forensic groups were significantly different in terms of gender and education: consequently, these variables were considered as possible confounders when assessing group differences on neurocognition and SC (Table 2). Moreover, also the influence of country of recruitment and age of first contact with mental health services was evaluated and no significant effect was found for these variables. Comparing unadjusted and adjusted results, education was found to be a confounder, and its confounding effect was very clear when assessing the relationship between the two groups with BACS and with SET. Moreover, education also affected the between-groups differences in some ER subscales (Table 2). After the adjustment for gender and education, only BACS Symbol-Coding (raw Cohen’s *d* = 0.46; adjusted *d* from estimated means = 0.41), ER Accuracy Fear (raw *d* = 0.18; adjusted *d* = 0.24), ER Accuracy Anger (raw *d* = 0.33; adjusted *d* = 0.35), ER Accuracy Contempt (raw *d* = 0.23; adjusted *d* = 0.37) and ER Total score (raw *d* = 0.28; adjusted *d* = 0.38) were significantly different between the two groups.

**Table 2:** Descriptive statistics of neurocognitive and social cognition tasks for forensic patients and controls.

	Forensic group *N* = 221 Mean (SD)	Control group *N* = 177 Mean (SD)	Cohen’s *d* effect size	Unadjusted *p* value	Adjusted *p* value
**BACS**					
List learning^a^^b^	32.6 (11.5)	35.6 (12.2)	0.25	**0.015**	0.311
Digits sequencing task^a^^b^	15.6 (4.7)	16.4 (4.8)	−0.17	0.177	0.560
Token motor task^a^^b^^c^	55.2 (16.7)	56.5 (16.6)	−0.08	0.662	0.978
Verbal fluency^a^^b^	36.6 (12.6)	39.7 (12.9)	−0.24	**0.021**	0.360
Symbol coding task^a^^b^	35.4 (12.9)	41.6 (14.1)	−0.46	**<0.001**	**0.003**
Tower of London^ab^	14.4 (5.2)	14.9 (5.1)	−0.1	0.320	0.785
Composite score^d^	−1.6 (1.0)	−1.3 (1.0)	−0.3	**0.007**	0.162
**Emotion recognition**					
Accuracy Surprise^a^	8.4 (2.0)	8.4 (1.9)	0	0.878	0.793
Accuracy Happiness^a^	9.8 (0.8)	9.6 (1.4)	0.18	0.435	0.494
Accuracy Fear^a^	5.1 (2.8)	4.6 (2.8)	0.18	0.117	**0.011**
Accuracy Disgust^a^	5.7 (2.8)	5.6 (2.8)	0.04	0.686	0.454
Accuracy Anger^a^	6.5 (2.3)	5.7 (2.5)	0.33	**0.007**	**0.005**
Accuracy Sadness^a^	6.7 (2.1)	6.4 (2.3)	0.14	0.327	0.079
Accuracy Contempt^a^	4.2 (3.5)	3.4 (3.4)	0.23	0.065	**<0.001**
Accuracy Neutral	9.1 (1.8)	8.8 (2.1)	0.15	0.059	0.320
Total score^a^	55.4 (9.9)	52.5 (11.0)	0.28	**0.041**	**<0.001**
**Story-based empathy task**					
SET GS^a^	13.8 (3.2)	14.5 (3.3)	−0.22	**0.013**	0.099
SET EA^a^	4.5 (1.4)	4.8 (1.4)	−0.21	**0.016**	0.192
SET IA^a^	4.7 (1.5)	5.1 (1.1)	−0.30	**0.010**	0.136
SET CI^a^	4.6 (1.1)	4.6 (1.3)	0	0.654	0.757

*BACS* Brief Assessment of Cognition in Schizophrenia.

^a^Means and standard deviations have been evaluated considering only valid cases (i.e., all cases with no missing data).

*d*: Cohen’s *d* effect size (forensic group – control group; *d* ≤ 0.2 small effect size; 0.2 < *d* ≤ 0.5 small-medium; 0.5 < *d* ≤ 0.8 medium-large; *d* ≥ 0.8 very large effect size).

Unadjusted *p* values have been obtained by using Mann–Whitney non-parametric test for BACS Digits sequencing task, Token motor task and Tower of London, for all ER scores and for all SET scores and *t*-test for BACS List learning, Verbal Fluency, Symbol coding task and Composite score.

Adjusted *p* values have been evaluated performing linear or generalized linear models adjusted for gender and education years.

^b^Raw scores.

^c^Data from the Polish sample for this test were excluded because of apparent administration errors leading to implausible scores.

^d^Composite score obtained from *z*-scores.

Bold values indicates statistically significant *p* values.

**Fig. 1:**
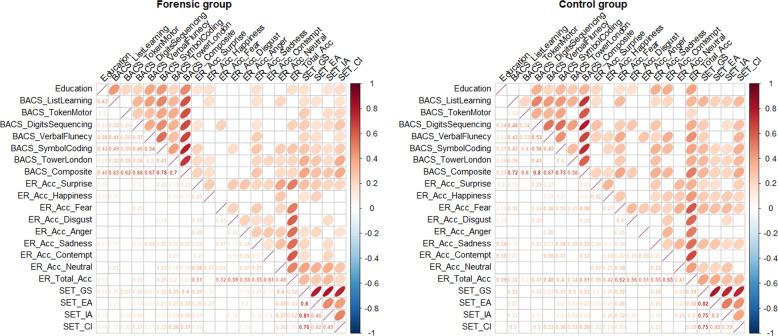
Correlation plots. Correlations among education, BACS, ER and set scores in the forensic (left panel) and in the control (right panel) groups.

The mutual relationships between SC, neurocognition and education are shown in Fig. 1. The correlation plots show only the significant correlations: almost all the variables were highly correlated (Spearman’s rho coefficients larger than 0.19 for the forensic group and larger than 0.17 for the control group, and rho coefficients larger than 0.35 among different domains of the same scale) and with similar significance in both forensic and non-forensic patients.

Given the results of the correlational analyses, logistic regression models were performed to investigate whether BACS scores affected the relationship between SC and groups (Supplementary Table 4S). The results of this analysis show that performance on the BACS accounts for the majority of the relationship between SET and the two groups. In addition, it has an effect on the relationship between the groups and ER Accuracy for Fear, ER Accuracy for Sadness and ER Accuracy for Contempt.

**Fig. 2:**
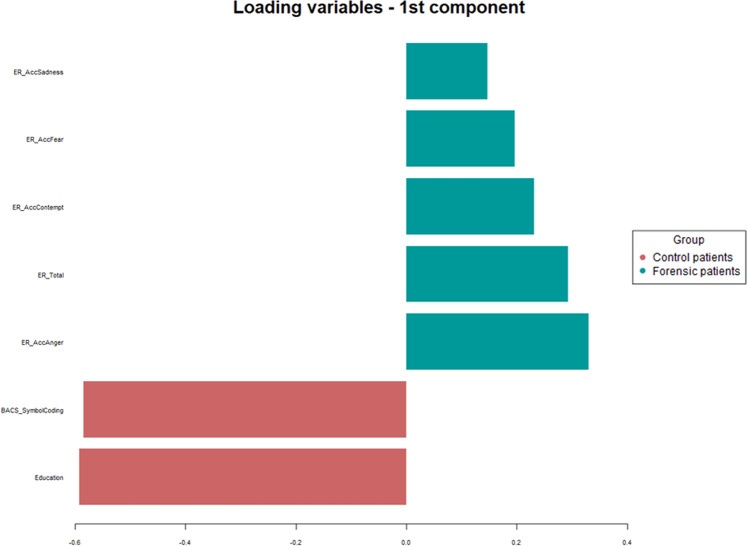
Partial least-squares discriminant analysis outputs. Loadings representing the contribution of each variable in discriminating forensic and controls groups.

Based on these results, all variables that were significantly different between the two groups, adjusted for education and gender (see Table 2), were analysed using PLS-DA, together with ER Accuracy for Sadness (that resulted significant in the logistic regression models—see Supplementary Table 4S). The outcome of this analysis is shown in the loading plot of Fig. 2, wherein the longer the bar, the higher the loading. Thus, through this analysis is possible to obtain a ranking of those variables which best discriminated between the two groups. Moreover, the colour of each variable bar indicates the group for which the mean value is higher. It can be noticed that education and BACS Symbol coding are the strongest discriminators of forensic and non-forensic patients, followed by ER scores. In particular, among ER scores, accuracy on anger recognition was the ER variable with highest loading.

Abnormal processing of anger in forensic patients was also suggested by the analysis of misidentification patterns in emotion recognition. The results (Supplementary Table 5S) show that non-forensic patients were more likely than forensic ones to misclassify anger as happiness, and contempt as fear. There was a stronger tendency to misclassify both surprise and fear as contempt in forensic patients. However, the most robust group difference in this respect concerns the stronger tendency to confuse disgust with anger in forensic, compared with non-forensic patients, with these results suggesting that the forensic cases identified more faces as showing contempt and anger than the control group.

## Discussion

This multinational study was aimed at examining the differential predictive potential of neurocognition and SC to discriminate violent forensic vs non-violent participants with SSD. Neurocognitive deficits have been recognized as a part of the fundamental disturbances in people with SSD and are a major determinant of functional outcome in this clinical population. Data exploring the link between neuropsychological deficits and the risk of violence in schizophrenia have been inconsistent [31, 32]. A recent meta-analysis found that only global cognitive impairment and lack of insight predicted violence in schizophrenia [33]. However, a recent study on 123 forensic patients with schizophrenia found that the relationship between cognition and violence was largely mediated by the social cognitive domain of the MATRICS Consensus Cognitive Battery [34] (MSCEIT). SC was also previously found to have a direct effect on violence, violence proneness and symptom severity independent of neurocognition [14]. Nevertheless, the few available studies on the role of empathy-related abilities (including ER and Theory of Mind, ToM) and the risk for violence in people with severe mental disorders provided inconsistent results. For example, violent patients with SSD were found not to be impaired in facial ER compared to controls, both in terms of responses time and accuracy [35]. On the other hand, results from a systematic review showed that violent patients with schizophrenia perform worse than HCs on ToM and ER tasks but outperform their controls with schizophrenia and no history of violence [36].

### Processing speed in people with SSD

Regarding neurocognition the only task in which forensic cases and controls showed a difference in performance was the BACS-Symbol Coding Task, a measure of processing speed. Processing speed reflects the speed at which different cognitive operations can be executed [37]. This finding is of special interest, because several studies have demonstrated that this simple measure can better discriminate people with SSD than other neuropsychological instruments [38–40], and is particularly sensitive to patients’ functional outcome [40, 41]. The Symbol Coding Task requires a quick and brief administration (approximately 5 min) and it is sensitive to a variety of developmental and clinical conditions [42]. Altered speed processing is a vulnerability-related component among relatives [43] and it may be present prior to illness onset [44]. Many studies in clinical populations have demonstrated that this task is a sensitive measure of cognitive dysfunction [37–39, 45], but a lack of specificity has also been reported [46] and deficits in processing speed are also common in bipolar disorder [47] and major depression [48]. Indeed, the performance of Symbol Coding Task involves a variety of cognitive operations, such as visual scanning, attention shifting, memory and motor speed, and the contribution of each of these cognitive abilities to the impairment on coding tasks is unclear and difficult to isolate [46]. Considering these findings, the use of this task has limitations for identification of specific cognitive deficits, but at the same time its multifaceted nature makes it highly sensitive for the detection of cognitive dysfunctions [39, 46]. Most studies using a measure of processing speed as a screening instrument for cognitive impairments in psychiatric patients involved SSD patients, with decreased processing speed being extensively reported [37–39, 49]. In a recent cohort study involving patients with severe mental disorders (*N* = 247 patients with SSD), where approximately half had a history of violence and the other half was never violent, we found that patients with an history of violence and who committed new acts of violence during the 1-year follow-up showed poorer performance on the Symbol Coding Task compared to non-violent patients [50].

In the literature on SSD several studies have suggested that processing speed deficits might be related to abnormalities in the connectivity of the cortical cerebellar–thalamic–cortical circuit, which may then affect higher-order processing capacities and lead to functional disturbances [38, 51, 52]. Other studies reported reduced grey matter volumes in bilateral prefrontal cortex, temporal lobe and superior temporal gyrus associated to a lower performance in symbol coding task [53]. Therefore, this area of research should be expanded and integrated with results of imaging studies.

### Social cognition in people with SSD and its relationship with violent behaviour

Both patient groups performed worse than HCs on the emotion recognition task; however, only non-forensic patients had significant lower scores than healthy subjects after adjustment for gender and education, particularly when attempting to correctly identify negative emotions such as fear and contempt. While confirming previous evidence impaired emotion recognition found in patients with SSD [54–60], associated with limbic neural structural [61] and functional [62, 63] alterations, this finding provides a reference for assessing in more depth emotion recognition skills between the two patient samples.

Alongside BACS symbol coding, augmented accuracy in recognizing one specific negative emotion—anger—was one of the strongest discriminant variables between forensic and non-forensic patients. In line with previous proposals about the importance of SC in mental disorders [64], this finding supports the role of anger processing as a critical marker of social cognitive functioning in conditions characterized by marked behavioural alterations and/or altered fronto-limbic brain activity or structure [65]. Defective performance in tasks revolving around the processing of potential threats, such as avoidance learning, aversive conditioning and both fear and anger processing, is indeed a typical hallmark of psychopathic traits, associated with structural [66] and functional [67] alterations involving the amygdala and its connections with the ventromedial prefrontal cortex. These brain structures are known to underpin different aspects of socio-affective processes, ranging from passive exposure and active processing of facial emotional expressions to downregulation of negative emotions [68]. Importantly, the present findings do not show defective anger recognition per se in forensic patients, who rather displayed better performance than controls with this emotion. When assessing the misidentification patterns in emotion recognition, however, the misclassification of disgust as anger represented the strongest discriminator between forensic and non-forensic patients (Supplementary Table 3S). At a deeper level of analysis than emotion recognition in itself, this evidence is suggestive of hypersensitivity to anger as a prominent driver of abnormal visual processing of negative emotions in forensic patients, possibly explaining their better performance in recognizing this emotion compared with non-forensic ones as being part of a response bias toward seeing anger in facial expressions. This might also explain the findings related to a socially oriented negative emotion such as *contempt* [69]. Also in this case, indeed, we observed impaired performance only in non-forensic patients compared with both forensic patients and HCs, while the latter groups did not differ with each other. This pattern fits with previous evidence of impaired contempt recognition in different samples of patients with schizophrenia [70, 71], but not in violent offenders with SSD [72]. As for anger, this pattern suggests that hypersensitivity to the facial expression of contempt differentiates these emotions, increasing the ability to recognize them despite a general pattern of impaired recognition of negative emotions. It is thus remarkable that such pattern involves anger and contempt, which have been labelled as “other-condemning” emotions for their inherently socio-moral orientation, embodying negative feelings about others’ actions or character and promoting decreased warmth, respect and compassion in social interaction [73].

In the light of this evidence on emotion recognition skills, it is notable that we did not find significant differences between forensic and non-forensic patients with regard to cognitive or affective mentalizing. While forensic patients might be expected to show the worst performance in this kind of task, some studies show that violent patients with schizophrenia outperform their ill controls with no history of violence in tasks tapping mentalizing skills [36]. Overall, this negative finding suggests that more sensitive—and possibly “ecological”—tools might be required to assess in-depth mentalizing and/or empathy skills in forensic populations.

### Limitations

Our study has a few limitations which should be acknowledged. First, scores in cognitive tasks might be affected by the pharmacological treatments, which may modulate cognitive performances of the groups. Selection of cases in studies such as this is always biased by refusal of participants to be involved in the research. The rates of refusal did not differ across the groups but we are unable to determine the reasons for refusal, which could be different. There are other social cognitive domains that could have been examined, including emotional regulation (e.g., via tasks assessing the implicit attentional capture by emotional stimuli, such as the emotional variant of the Stroop task [74]). The latter however requires personal computers for recording response time, and the constraints related to the conduct of this study included the prohibition to introduce computer or tablet in forensic units. In the trade-off between comprehensiveness and feasibility, however, we opted for the emotional recognition task, which was found to have the best psychometric properties in previous studies [9, 75]. HCs were fewer in number and not selected for demographic similarity to SSD participants.

### Concluding remarks

Our study provides robust data which expand our knowledge about neurocognition and SC in people with SSD and provide hints to better understand some of the multidimensional pathways (neurobiological, clinical and environmental) which drive violent behaviour in a limited number of people with psychosis. They have important clinical implications to improve the multidimensional assessment of forensic patients with SSD, who may benefit from an appropriate social cognitive and neurocognitive assessment, so far largely missed in ordinary clinical practice. Treatment of social cognitive and neurocognitive deficits has been proven to reduce violent behaviour, so implementation of these interventions seems to be a reasonable evidence-based practice suggestion.

## Supplementary information

LIST OF FORENSIC FACILITIES RECRUITING FOR THE EU-VIORMED PROJECT

BACS Z-SCORES

EMOTION RECOGNITION ACCURACY IN FORENSIC, NON-FORENSIC PATIENTS AND HEALTHY ADULTS

UNIVARIATE AND MULTIPLE LOGISTIC MODELS: ASSOCIATION BETWEEN SOCIAL COGNITIVE VARIABLES (INDEPENDENT VARIABLES) AND THE TWO GROUPS (FORENSIC AND CONTROL GROUP) UNADJUSTED AND ADJUSTED

MISIDENTIFICATION PATTERNS OF THE EMOTION RECOGNITION TEST

## Data Availability

The project will fully embrace the open access data policy of H2020 to make data FAIR (Findable, Accessible, Interoperable, and Re-usable), and all data gathered in the framework of the project are stored in a public repository (10.5281/zenodo.4442372) accessible to all scientists willing to carry out additional analyses.
