# Supplementary material for: Neurocognition and social cognition in patients with schizophrenia spectrum disorders with and without a history of violence: results of a multinational European study
Source: Transl Psychiatry. 2021 Dec 8;11:620. doi: 10.1038/s41398-021-01749-1 (PMC8651972; doi:10.1038/s41398-021-01749-1)
Supplement: Supplementary file 1 — LIST OF FORENSIC FACILITIES RECRUITING FOR THE EU-VIORMED PROJECT [file 41398_2021_1749_MOESM1_ESM.docx]

**Supplementary materials**

**TABLE 1 SUPPLEMENTARY**

**LIST OF FORENSIC FACILITIES RECRUITING FOR THE EU-VIORMED PROJECT**

| COUNTRY | FACILITY NAME AND LOCATION | NUMBER OF FORENSIC BEDS | OFFICIAL SECURITY LEVEL |
| --- | --- | --- | --- |
| AUSTRIA | Justizanstalt Goellersdorf, Lower Austria, Goellersdorf | 145 | NA |
|  | Klinik für Psychiatrie mit forensischem Schwerpunkt, Upper Austria, Linz | 53 | NA |
| GERMANY | Klinik für Forensische Psychiatrie und Psychotherapie, Zentrum für Psychiatrie Nordbaden, Wiesloch | 258 | NA |
|  | Klinik für Forensische Psychiatrie des Pfalzklinikums, Klingenmünster | 185 | NA |
|  | Klinik für Forensische Psychiatrie und Psychotherapie, Weinsberg | 124 | NA |
| ITALY | REMS, Centro Polifunzionale ex Ospedale Stellini, ULSS 9 ‘Scaligera’, Nogara | 130 | NA |
|  | Sistema Polimodulare REMS, Castiglione delle Stiviere | 20 | NA |
|  | REMS Minerva, ASL Roma 5, Rome | 20 | NA |
|  | REMS-D, Azienda USL Toscana Nord-Ovest, Volterra | 20 | NA |
| POLAND | Institute of Psychiatry and Neurology, Department of Forensic Psychiatry, Warsaw | 60 | Medium |
|  | SP ZOZ Psychiatryc Hospital, Department of Forensic Psychiatry, Toszek, | 40 | Medium |
|  | Psychiatric Hospital, Forensic Department, Starogard Gdański | 40 | Low |
| UNITED KINGDOM | South London and Maudsley NHS Foundation Trust, London | 130 | Low and Medium |
|  | South West London and St. Georges Mental Health NHS Trust, London | 75 | Medium |
|  | St. Andrew’s Healthcare | 240 | Low and Medium |

NA : Not Applicable, because there are no different security levels.
