# Supplementary material for: Neurocognition and social cognition in patients with schizophrenia spectrum disorders with and without a history of violence: results of a multinational European study
Source: Transl Psychiatry. 2021 Dec 8;11:620. doi: 10.1038/s41398-021-01749-1 (PMC8651972; doi:10.1038/s41398-021-01749-1)
Supplement: Supplementary file 2 — BACS Z-SCORES [file 41398_2021_1749_MOESM2_ESM.docx]

**Supplementary materials**

**TABLE 2 SUPPLEMENTARY**

**BACS Z-SCORES**

|  | **Forensic group**  **N= 221**  **Mean (SD)** | **Control group**  **N=177**  **Mean (SD)** | **Cohen’s d effect size** | **p-value** |
| --- | --- | --- | --- | --- |
| **BACS** |  |  |  |  |
| *List learning** | -1.6 (1.3) | -1.3 (1.5) | -0.22 | **0.029** |
| *Digit sequencing** | -1.8 (1.4) | -1.5 (1.3) | -0.22 | 0.101 |
| *Token motor** | -1.3 (1.4) | -1.1 (1.5) | -0.14 | 0.285 |
| *Verbal fluency** | -1.5 (1.1) | -1.2 (1.1) | -0.27 | 0.103 |
| *Symbol coding** | -2.2 (1.1) | -1.7 (1.2) | -0.44 | **<0.001** |
| *Tower of London** | -1.0 (1.8) | -0.9 (1.6) | -0.06 | 0.406 |
| *Composite score** | -1.6 (1.0) | -1.3 (1.0) | -0.30 | **0.007** |

BACS: Brief Assessment of Cognition in Schizophrenia.

*Means and standard deviations have been evaluated considering only valid cases (i.e. all cases with no missing data).

Mann-Whitney non-parametric test has been performed.

d: Cohen’s d effect size (forensic group – control group; d<=0.2 small effect size; 0.2<d<=0.5 small-medium; 0.5<d<=0.8 medium-large; d>=0.8 very large effect size).
