# Supplementary material for: Neurocognition and social cognition in patients with schizophrenia spectrum disorders with and without a history of violence: results of a multinational European study
Source: Transl Psychiatry. 2021 Dec 8;11:620. doi: 10.1038/s41398-021-01749-1 (PMC8651972; doi:10.1038/s41398-021-01749-1)
Supplement: Supplementary file 3 — EMOTION RECOGNITION ACCURACY IN FORENSIC, NON-FORENSIC PATIENTS AND HEALTHY ADULTS [file 41398_2021_1749_MOESM3_ESM.docx]

**Supplementary materials**

**TABLE 3 SUPPLEMENTARY**

**EMOTION RECOGNITION ACCURACY IN FORENSIC, NON-FORENSIC PATIENTS AND HEALTHY ADULTS**

|  | **Forensic group (FG)**  **N=221**  **Mean (SD)** | **Control group (CG)**  **N=177**  **Mean (SD)** | **Healthy group (HG)**  **N=57**  **Mean (SD)** | **Unadjusted p-value** | **Unadjusted**  **Post-hoc** | **Adjusted p-value** | **Adjusted**  **Post-hoc** |
| --- | --- | --- | --- | --- | --- | --- | --- |
| **EMOTION RECOGNITION** |  |  |  |  |  |  |  |
| *Accuracy Surprise** | 8.4 (2.0) | 8.4 (1.9) | 9.2 (1.1) | **0.004** | (FG) vs (HG) 0.004 d=-0.43  (CG) vs (HG) 0.010 d=-0.46 | 0.397 |  |
| *Accuracy Happiness** | 9.8 (0.8) | 9.6 (1.4) | 10.0 (0.2) | 0.169 |  | 0.788 |  |
| *Accuracy Fear** | 5.1 (2.8) | 4.6 (2.8) | 6.3 (2.8) | **<0.001** | (FG) vs (HG) 0.011 d=-0.43  (CG) vs (HG) <0.001 d=-0.61 | **0.005** | (FG) vs (CG) 0.010  (CG) vs (HG) 0.004 |
| *Accuracy Disgust** | 5.7 (2.8) | 5.6 (2.8) | 7.0 (2.7) | **0.006** | (FG) vs (HG) 0.013 d=-0.47  (CG) vs (HG) 0.008 d=-0.50 | 0.067 |  |
| *Accuracy Anger** | 6.5 (2.3) | 5.7 (2.5) | 6.8 (2.2) | **0.005** | (FG) v (CG) 0.021 d=0.33  (CG) vs (HG) 0.018 d=-0.45 | **0.012** | (FG) vs (CG) 0.003 |
| *Accuracy Sadness** | 6.7 (2.1) | 6.4 (2.3) | 7.2 (1.7) | 0.213 |  | 0.291 |  |
| *Accuracy Contempt** | 4.2 (3.5) | 3.4 (3.4) | 4.9 (3.1) | **0.012** | (CG) vs (HG) 0.012 d=-0.45 | **<0.001** | (FG) vs (CG) <0.001  (CG) vs (HG) 0.012 |
| *Accuracy Neutral* | 9.1 (1.8) | 8.8 (2.1) | 9.6 (1.7) | **0.036** | (CG) vs (HG) 0.054 d=-0.40 | 0.437 |  |
| **Total score*** | 55.4 (9.9) | 52.5 (11.0) | 60.9 (8.4) | **<0.001** | (FG) vs (HG) 0.001 d=-0.57  (CG) vs (HG) <0.001 d=-0.81 | **<0.001** | (FG) vs (CG) <0.001  (CG) vs (HG) <0.001 |

*Means and standard deviations have been evaluated considering only valid cases (i.e. all cases with no missing data).

d: Cohen’s d effect size for observed (unadjusted) data (forensic group – control group; d<=0.2 small effect size; 0.2<d<=0.5 small-medium; 0.5<d<=0.8 medium-large; d>=0.8 very large effect size).

Unadjusted p-values and post-hoc have been obtained by using Kruskal-Wallis non-parametric test.

Adjusted p-values and post-hoc have been evaluated performing generalized linear models adjusted for gender and education years.
