# Supplementary material for: Neurocognition and social cognition in patients with schizophrenia spectrum disorders with and without a history of violence: results of a multinational European study
Source: Transl Psychiatry. 2021 Dec 8;11:620. doi: 10.1038/s41398-021-01749-1 (PMC8651972; doi:10.1038/s41398-021-01749-1)
Supplement: Supplementary file 4 — UNIVARIATE AND MULTIPLE LOGISTIC MODELS: ASSOCIATION BETWEEN SOCIAL COGNITIVE VARIABLES (INDEPENDENT VARIABLES) AND THE TWO GROUPS (FORENSIC AND CONTROL GROUP) UNADJUSTED AND ADJUSTED [file 41398_2021_1749_MOESM4_ESM.docx]

**Supplementary materials**

**TABLE 4 SUPPLEMENTARY**

**UNIVARIATE AND MULTIPLE LOGISTIC MODELS: ASSOCIATION BETWEEN SOCIAL COGNITIVE VARIABLES (INDEPENDENT VARIABLES) AND THE TWO GROUPS (FORENSIC AND CONTROL GROUP) UNADJUSTED AND ADJUSTED FOR BACS COMPOSITE SCORE, SEX AND EDUCATION.**

| **Model** | **Independent Variables** | **OR [95%CI]**  **(unadjusted model)** | **p-value**  **(unadjusted model)** | **AIC**  **(unadjusted model** | **OR [95%CI] (adjusted model)** | **p-value (adjusted model)** | **AIC**  **(adjusted model)** |
| --- | --- | --- | --- | --- | --- | --- | --- |
| A | ER Total Score | 1.34 [1.12-1.58] | **0.018** | 390.81 |  |  |  |
| A.1 | ER Total Score | 1.59 [1.52-1.68] | **0.001** | 365.64 | 1.75 [1.33-2.89] | **<0.001** | 357.88 |
|  | BACS Composite Score | 0.55 [0.53-0.58] | **<0.001** |  | 0.64 [0.59-0.82] | **0.005** |  |
| B | ER Accuracy Surprise | 0.99 [0.70-2.95] | 0.947 | 396.55 |  |  |  |
| B.1 | ER Accuracy Surprise | 1.05 [0.73- 2.76] | 0.734 | 377.48 | 1.07 [0.67-1.59] | 0.628 | 373.32 |
|  | BACS Composite Score | 0.64 [0.57- 0.75] | **0.002** |  | 0.73 [0.59-0.94] | **0.039** |  |
| C | ER Accuracy Happiness | 1.21 [0.87-1.43] | 0.144 | 394.18 |  |  |  |
| C.1 | ER Accuracy Happiness | 1.26 [0.85-1.38]] | 0.124 | 375.08 | 1.31 [0.96- 1.56] | 0.076 | 370.16 |
|  | BACS Composite Score | 0.63 [0.56-0.67] | **0.001** |  | 0.72 [0.57-0.89] | **0.033** |  |
| D | ER Accuracy Fear | 1.21 [0.92-1.39] | 0.110 | 393.98 |  |  |  |
| D.1 | ER Accuracy Fear | 1.35 [1.11-1.59] | **0.019** | 371.96 | 1.39 [1.18-1.60] | **0.012** | 367.01 |
|  | BACS Composite Score | 0.60 [0.53-0.65] | **0.001** |  | 0.69 [0.49-0.89] | **0.019** |  |
| E | ER Accuracy Disgust | 1.04 [0.79-1.26] | 0.732 | 396.43 |  |  |  |
| E.1 | ER Accuracy Disgust | 1.13 [0.88-1.33] | 0.319 | 376.60 | 1.15 []0.79-1.30] | 0.267 | 372.32 |
|  | BACS Composite Score | 0.63 [0.57-0.70] | **0.001** |  | 0.72 [0.52-0.92] | **0.032** |  |
| F | ER Accuracy Anger | 1.38 [1.21-1.51] | **0.008** | 389.26 |  |  |  |
| F.1 | ER Accuracy Anger | 1.47 [1.28-1.62] | **0.002** | 367.96 | 1.52 [1.31-1.78] | **0.001** | 362.87 |
|  | BACS Composite Score | 0.63 [0.55-0.67] | **0.001** |  | 0.72 [0.52-0.92] | **0.040** |  |
| G | ER Accuracy Sadness | 1.15 [0.94-1.20] | 0.233 | 395.12 |  |  |  |
| G.1 | ER Accuracy Sadness | 1.31 [1.04-1.68] | **0.041** | 373.32 | 1.41 [1.22-1.69] | **0.014** | 367.28 |
|  | BACS Composite Score | 0.59 [0.54-0.61] | **<0.001** |  | 0.67 [0.55-0.93] | **0.013** |  |
| H | ER Accuracy Contempt | 1.26 [0.97-1.15] | 0.060 | 392.96 |  |  |  |
| H.1 | ER Accuracy Contempt | 1.39 [1.10-1.88] | **0.011** | 370.84 | 1.48 [1.31-1.78] | **0.004** | 364.87 |
|  | BACS Composite Score | 0.60 [0.57-0.64] | **<0.001** |  | 0.70 [0.50-0.90] | **0.020** |  |
| I | ER Accuracy Neutral | 1.15 [0.76-1.48] | 0.252 | 395.23 |  |  |  |
| I.1 | ER Accuracy Neutral | 1.16 [0.81-1.45] | 0.245 | 376.22 | 1.20 [0.95-1.54] | 0.151 | 371.47 |
|  | BACS Composite Score | 0.64 [0.55-0.75] | **0.002** |  | 0.73 [0.47-0.96] | **0.043** |  |
| J | SET GS | 0.77 [0.63-91] | **0.025** | 460.82 |  |  |  |
| J.1 | SET GS | 0.89 [0.57-1.34] | 0.376 | 441.65 | 0.89 [0.47-1.38] | 0.385 | 437.00 |
|  | BACS Composite Score | 0.68 [0.59-0.83] | **0.006** |  | 0.77 [0.53-1.19] | 0.087 |  |
| K | SET EA | 0.77 [0.45-0.90] | **0.025** | 458.67 |  |  |  |
| K.1 | SET EA | 0.86 [0.66-1.18] | 0.233 | 439.42 | 0.86 [0.43-1.22] | 0.235 | 434.68 |
|  | BACS Composite Score | 0.69 [0.53-0.84] | **0.005** |  | 0.78 [0.49-1.10] | 0.095 |  |
| L | SET IA | 0.72 [0.59-0.83] | **0.006** | 456.92 |  |  |  |
| L.1 | SET IA | 0.79 [0.57-1.02] | 0.054 | 437.89 | 0.79 [0.54-1.04] | 0.067 | 433.51 |
|  | BACS Composite Score | 0.70 [0.58- 0.82] | **0.007** |  | 0.78 [0.51-1.09] | 0.092 |  |
| M | SET CI | 0.99 [0.67-1.28] | 0.959 | 464.99 |  |  |  |
| M.1 | SET CI | 1.21 [0.59- 1.57] | 0.136 | 439.50 | 1.21 [0.92-1.48] | 0.144 | 434.85 |
|  | BACS Composite Score | 0.60 [0.57-0.64] | **<0.001** |  | 0.68 [0.46-0.88] | **0.013** |  |

ER: Emotion Recognition; BACS: Brief Assessment of Cognition in Schizophrenia; SET: Story of Empathy Task.

Reference category=Control group.

Standardized scores have been used for ER and SET. All the models adjusted for BACS have also been adjusted for gender, education.
