# Supplementary material for: Neurocognition and social cognition in patients with schizophrenia spectrum disorders with and without a history of violence: results of a multinational European study
Source: Transl Psychiatry. 2021 Dec 8;11:620. doi: 10.1038/s41398-021-01749-1 (PMC8651972; doi:10.1038/s41398-021-01749-1)
Supplement: Supplementary file 5 — MISIDENTIFICATION PATTERNS OF THE EMOTION RECOGNITION TEST [file 41398_2021_1749_MOESM5_ESM.docx]

**Supplementary materials**

**TABLE 5 SUPPLEMENTARY**

**MISIDENTIFICATION PATTERNS OF THE EMOTION RECOGNITION TEST**

|  | **EMOTION CONFUSED WITH** | | | | | | | | | | | | | | | |
| --- | --- | --- | --- | --- | --- | --- | --- | --- | --- | --- | --- | --- | --- | --- | --- | --- |
|  | **SURPRISE** | | **HAPPINESS** | | **FEAR** | | **DISGUST** | | **ANGER** | | **SADNESS** | | **CONTEMPT** | | **NEUTRAL** | |
| **Emotion presented** | N (%) * | p | N (%) * | p | N (%) * | p | N (%) * | p | N (%) * | p | N (%) * | p | N (%) * | p | N (%) * | p |
| **SURPRISE** |  |  |  |  |  |  |  |  |  |  |  |  |  |  |  |  |
| *Controls* | - | - | 13 (10.5) | 0.353 | 38 (30.6) | 0.231 | 16 (12.9) | 0.259 | 11 (8.9) | 0.641 | 7 (5.6) | 0.343 | 17 (13.7) | **0.031** | 10 (8.1) | 0.735 |
| *Forensic pts.* | - |  | 12 (7.4) |  | 61 (37.4) |  | 29 (17.8) |  | 12 (7.4) |  | 14 (8.6) |  | 39 (23.9) |  | 15 (9.2) |  |
| **HAPPINESS** |  |  |  |  |  |  |  |  |  |  |  |  |  |  |  |  |
| *Controls* | 9 (7.3) | 0.548 | - | - | 0 (0.0) | 1.000 | 1 (0.8) | 0.432 | 3 (2.4) | 0.080 | 0 (0.0) | ---# | 1 (0.8) | 0.636 | 4 (3.2) | 0.354 |
| *Forensic pts.* | 9 (5.5) |  | - |  | 1 (0.6) |  | 0 (0.0) |  | 0 (0.0) |  | 0 (0.0) |  | 3 (1.8) |  | 9 (5.5) |  |
| **FEAR** |  |  |  |  |  |  |  |  |  |  |  |  |  |  |  |  |
| *Controls* | 89 (71.8) | 0.821 | 9 (7.3) | 0.054 | - | - | 86 (69.4) | 0.820 | 30 (24.2) | 0.495 | 32 (25.8) | 0.737 | 26 (21.0) | **0.028** | 12 (9.7) | 0.955 |
| *Forensic pts.* | 115 (70.6) |  | 4 (2.5) |  | - |  | 111 (68.1) |  | 45 (27.8) |  | 39 (24.1) |  | 53 (32.7) |  | 16 (9.9) |  |
| **DISGUST** |  |  |  |  |  |  |  |  |  |  |  |  |  |  |  |  |
| *Controls* | 20 (16.1) | 0.283 | 4 (3.2) | 0.762 | 11 (8.9) | 0.434 | - | - | 85 (68.5) | **<0.001** | 13 (10.5) | 0.473 | 67 (54.0) | 0.079 | 12 (9.7) | 0.071 |
| *Forensic pts.* | 19 (11.7) |  | 7 (4.3) |  | 19 (11.7) |  | - |  | 143 (87.7) |  | 13 (8.0) |  | 71 (43.6) |  | 7 (4.3) |  |
| **ANGER** |  |  |  |  |  |  |  |  |  |  |  |  |  |  |  |  |
| *Controls* | 26 (21.0) | 0.881 | 7 (5.6) | **0.023** | 27 (21.8) | 0.327 | 54 (43.5) | 0.678 | - | - | 46 (37.1) | 0.138 | 68 (54.8) | 0.867 | 57 (46.0) | 0.842 |
| *Forensic pts.* | 33 (20.2) |  | 1 (0.6) |  | 28 (17.2) |  | 67 (41.1) |  | - |  | 47 (28.8) |  | 91 (55.8) |  | 73 (44.8) |  |
| **SADNESS** |  |  |  |  |  |  |  |  |  |  |  |  |  |  |  |  |
| *Controls* | 34 (27.4) | 0.311 | 4 (3.2) | 0.170 | 57 (46.0) | 0.569 | 35 (28.2) | 0.094 | 53 (42.7) | 0.484 | - | - | 46 (37.1) | 0.211 | 44 (35.5) | 0.201 |
| *Forensic pts.* | 36 (22.2) |  | 1 (0.6) |  | 69 (42.6) |  | 61 (37.7) |  | 63 (38.7) |  | - |  | 72 (44.4) |  | 46 (28.4) |  |
| **CONTEMPT** |  |  |  |  |  |  |  |  |  |  |  |  |  |  |  |  |
| *Controls* | 37 (29.8) | 0.086 | 35 (28.2) | 0.559 | 14 (11.3) | **0.046** | 36 (29.0) | 0.052 | 18 (14.5) | 0.390 | 33 (26.6) | 0.803 | - | - | 100 (80.6) | 0.493 |
| *Forensic pts.* | 34 (21.0) |  | 41 (25.2) |  | 8 (4.9) |  | 65 (40.1) |  | 18 (11.1) |  | 41 (25.3) |  | - |  | 126 (77.3) |  |
| **NEUTRAL** |  |  |  |  |  |  |  |  |  |  |  |  |  |  |  |  |
| *Controls* | 17 (13.7) | 0.166 | 12 (9.7) | 0.493 | 7 (5.6) | 0.599 | 5 (4.0) | 1.000 | 6 (4.8) | 0.797 | 17 (13.7) | 0.699 | 21 (16.9) | 0.850 | - | - |
| *Forensic pts.* | 14 (8.6) |  | 12 (7.4) |  | 7 (4.3) |  | 6 (3.7) |  | 9 (5.5) |  | 25 (15.3) |  | 29 (17.8) |  | - |  |

Frequencies and percentages have been evaluated considering only valid cases (i.e. all cases with no missing data). Binomial proportion test has been performed for all comparisons.
